# Steric Stabilization of “Charge-Free” Cellulose Nanowhiskers by Grafting of Poly(ethylene glycol)

**DOI:** 10.3390/molecules20010169

**Published:** 2014-12-24

**Authors:** Jun Araki, Shiho Mishima

**Affiliations:** 1 Faculty of Textile Science and Technology, Shinshu University, Tokida 3-15-1, Ueda, Nagano Prefecture 386-8567, Japan; 2 Division of Biological and Medical Fibers, Institute for Fiber Engineering (IFES), Interdisciplinary Cluster for Cutting Edge Research (ICCER), Shinshu University, Tokida 3-15-1, Ueda, Nagano Prefecture, 386-8567, Japan; 3 Graduate School of Science and Technology, Shinshu University, Tokida 3-15-1, Ueda, Nagano Prefecture, 386-8567, Japan

**Keywords:** steric stabilization, cellulose nanowhiskers, poly(ethylene glycol), 1,1'-carbonyldiimidazole (CDI), dispersion stability

## Abstract

A sterically stabilized aqueous suspension of “charge-free” cellulose nanowhiskers was prepared by hydrochloric acid hydrolysis of cotton powders and subsequent surface grafting of monomethoxy poly(ethylene glycol) (mPEG). The preparation scheme included carboxylation of the terminal hydroxyl groups in mPEG via oxidation with silica gel particles carrying 2,2,6,6-tetramethyl-1-pyperidinyloxyl (TEMPO) moieties and subsequent esterification between terminal carboxyls in mPEG and surface hydroxyl groups of cellulose nanowhiskers, mediated by 1,1'-carbonyldiimidazole (CDI) in dimethyl sulfoxide or dimethylacetamide. Some of the prepared PEG-grafted samples showed remarkable flow birefringence and enhanced stability after 24 h, even in 0.1 M NaCl, suggesting successful steric stabilization by efficient mPEG grafting. Actual PEG grafting via ester linkages was confirmed by attenuated total reflectance-Fourier transform infrared spectrometry. In a typical example, the amount of grafted mPEG was estimated as *ca.* 0.3 g/g cellulose by two measurements, *i.e.*, weight increase after grafting and weight loss after alkali cleavage of ester linkages. Transmission electron microscopy indicated unchanged nanowhisker morphology after mPEG grafting.

## 1. Introduction

It is known that stable, non-sedimenting aqueous suspensions of rod-like crystalline particles can be prepared by appropriate treatments of native cellulose or chitin with mineral acids [1,2,3,4]. These colloidal particles, which have recently been called “nanowhiskers” of these polysaccharides, are shortened fragments of bio-synthesized “microfibrils” [5], in which linear molecules of the polysaccharides are aligned in parallel to form long-ranging fine fibers. While the original microfibrils have lengths of an order exceeding tens of micrometers, exceeding areas of microscopic observations, nanowhiskers usually comprise shorter length scales. For example, lengths are 100–300 nm for cellulose nanowhiskers derived from higher plants and *α*-chitin nanowhiskers from crab shells [6,7,8,9,10,11], 1–2 µm for those from bacterial cellulose [12] and up to several micrometers for nanowhiskers from tunicins or green algae [13,14]. The widths of nanowhiskers also vary according to their origin, namely, 2–10 nm for cellulose nanowhiskers from higher plants and crab chitin nanowhiskers, and up to 20 nm for tunicin or algal cellulose nanowhiskers. The cross-sectional size of ribbon-like bacterial cellulose nanowhiskers is reported to be *ca.* 10 × 50 nm [12].

These nanowhiskers and their colloidal suspensions have been attracting significant attention due to their striking characteristics, one of which is their lyotropic liquid crystallinity. Above a critical concentration, suspensions of these nanowhiskers are reported to spontaneously separate into upper isotropic and lower anisotropic phases, the latter containing chiral nematic [8,9,10,11,12,15,16] or nematic [15] ordering. The formed liquid crystalline domains are also known to align their chiral nematic axis parallel to applied magnetic field to form long-ranging anisotropic domains [9,17]. Under some conditions, an unusual “birefringent glassy phase” was formed, irrespective of concentration [16].

Another intriguing feature of the nanowhiskers is their outstanding mechanical properties, *i.e.*, extremely high modulus and strength. The Young’s moduli of tunicin cellulose nanowhiskers and squid pen chitin nanowhiskers are both estimated as 150 GPa from a bending test using an atomic force microscope [18] and a calculation from the moduli of nanocomposites [19], respectively. These high values of modulus and strength are attributed to formation of an almost perfect single crystal by parallel alignment of rigid polysaccharide chains, which are bound to each other with many strong hydrogen bonds. Much attention has been focused recently on the utilization of these nanowhiskers as nano-sized fillers in composites, due to their excellent mechanical properties, as well as non-toxicity, biodegradability and ease of large-scale preparation. For example, in the last two decades there have been numerous reports on various types of nanowhisker composites, including films containing matrices of latex or synthetic polymers and fillers of nanowhiskers [19,20,21], high-modulus poly(vinyl alcohol) wet-spun fibers containing uniaxially oriented nanowhiskers [22,23,24,25], hydrogels reinforced by nanowhiskers [26,27] and electrospun fiber mats incorporating nanowhiskers [28].

Good dispersion of independent nanowhiskers in matrices is quite significant for the preparation of the above-mentioned nanocomposites, because well-dispersed nanofillers usually increase their specific surface area to enhance interactions with matrices and thereby improve mechanical properties, whereas aggregations of nanofillers often act as defects in nanocomposites or cause undesirable stress concentration resulting in a significant reduction in mechanical properties. Dispersion stability of the nanowhiskers is mainly dependent on repulsive forces caused by surface charge, *i.e.*, electrostatic stabilization. Negative charges of surface sulfate groups on cellulose nanowhiskers generated during sulfuric acid treatment [6,7,8,9] and positive charges of inherent surface amino groups on chitin nanowhiskers [10,11] are believed to be responsible for their stability. This electrostatic stability, however, was significantly reduced by addition of electrolytes via the shielding effect. Therefore, aqueous nanowhisker suspensions easily form aggregates to precipitate in the presence of electrolytes. The effect of electrostatic repulsion is also minor in media with low dielectric constants, because the range of electrostatic repulsion is proportional to the dielectric constant of the medium. Dispersion of nanowhiskers in organic solvents has not been achieved, except for those possessing high dielectric constants, such as dimethyl sulfoxide (DMSO) and dimethylformamide (DMF) [29].

Another approach to solve the problem of electrostatic stabilization described above is so-called “steric stabilization”, in which polymers grafted or adsorbed on particle surfaces prevent the approach of the particle to prevent aggregation, resulting in a stable dispersion [30]. In 2000 and 2001, two pioneering approaches to steric stabilization of cellulose nanowhiskers were examined by different research groups; one was preparation of cellulose nanowhiskers with surface-adsorbed surfactants, which gave a good dispersion stability in toluene or tetrahydrofuran [31,32], while the other was surface grafting of poly(ethylene glycol) (PEG) onto cellulose nanowhiskers [33,34]. Starting from these two researches, many literature articles have reported preparations of a wide variety of sterically stabilized nanowhisker suspensions, including ring opening living polymerization of °ε-caprolactone initiated by surface hydroxyls on cellulose nanowhiskers [35,36], introduction of polystyrene [37] or poly(*N*,*N*-dimethylaminoethyl methacrylate) [38] via atom transfer radical polymerization, single electron transfer living radical polymerization of *N*-isopropylacrylamide on whisker surfaces [39], and introduction of amine-terminated ethylene oxide-propylene oxide copolymers via amidation [40]. Steric stabilization of nanowhiskers derived from chitin, a polysaccharide consisting of crab/shrimp shells and having a linear structure similar to that of cellulose, were also realized very recently [41]. Although these samples can be useful for applications such as nanocomposite preparation, such a coexistence of steric and electrostatic stabilization may sometimes disturb a detailed investigation on a genuinely steric effect; namely, most of the above-mentioned studies use stable nanowhisker suspensions with surface charge groups, which remain after surface grafting and/or adsorption of polymers. When the carboxyl groups on nanowhiskers were used as the binding site for polymers, significant amounts of carboxyls remained unreacted and acted as surface charge groups [33]. As a result, stability of the obtained suspensions is dependent not only on the steric repulsions of surface polymers but also on the charge repulsions by residual charge groups. To independently evaluate the effect of grafted or adsorbed polymers on stability, preparation of nanowhiskers that are free of charge and possess surface polymers, is required. Kloser and Gray [42] first examined a preparation of such a sample by desulfation of nanowhiskers with 0.1 M NaOH treatment and subsequent grafting of poly(ethylene oxide), although very trace amount of sulfate groups (sulfur content of 0.04 mmol/g cellulose) could not be removed.

**Scheme 1:**
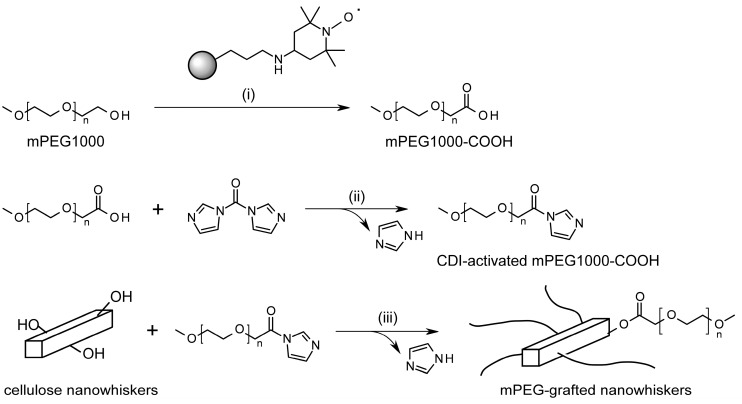
Procedures for mPEG1000-COOH grafting onto “charge-free” nanowhiskers via CDI-mediated esterification.

“Charge-free” cellulose nanowhiskers can be prepared by hydrolysis of native cellulose with hydrochloric acid and subsequent mechanical homogenization [33,43,44]. In the present study, the authors examined preparation of a sterically stabilized cellulose nanowhisker suspension according to Scheme 1, via esterification between surface hydroxyl groups of the “charge-free” nanowhiskers and terminal carboxyl groups of monomethoxy PEG (mPEG), mediated by 1,1'-carbonyldiimidazole (CDI).

*Reagents and Conditions*: (i) NaClO, NaBr, pH = 10–11, r.t., 1 h; (ii) DMAc or DMSO, r.t., 2 h; (iii) DMAc or DMSO, 80 or 100 °C, 1–7 day(s).

## 2. Results and Discussion

### 2.1. Strategy for PEG-Grafting onto “Charge-Free” Cellulose Nanowhiskers

As shown in Scheme 1, the authors examined a novel method for PEG grafting onto cellulose nanowhiskers via CDI-mediated esterification between surface hydroxyl groups of cellulose nanowhiskers and terminal carboxyl groups of mPEG1000 in the present study, instead of the previously used amidation between surface carboxyls of oxidized nanowhiskers and terminal amino groups of mPEG [33]. This CDI-mediated esterification was previously employed for other modifications, including binding of carboxyl derivatives to dissolved cellulose molecules [45] or modifications of hydroxyl groups in a polyrotaxane, *i.e.*, a supramolecule containing oligosaccharide moieties [46,47]. All of the previous studies reported successful formation of adducts under relatively mild reaction conditions. Another reason for application of the CDI-mediated esterification to the PEG grafting on cellulose nanowhiskers is no necessity of introduction of surface functional groups; an introduction of such groups (like carboxyls) requires one or more reaction steps before grafting, and may leave some unreacted groups, which further can generate a charge repulsion if they are ionic.

The starting suspension of “charge-free” nanowhiskers were prepared by hydrochloric acid hydrolysis of amorphous regions in CF11, a cotton-derived native cellulose sample, and subsequent homogenization using strong shear force and centrifugation processes, according to previous studies [6,7,33,44]. In contrast to the well-known hydrolysis process with sulfuric acid [6,7,8,9], no sulfation of surface hydroxyls occurs with the hydrochloric acid hydrolysis. However, the hydrochloric acid treatment yields aqueous suspensions of nanowhiskers, which are unsuitable for the subsequent esterification mediated with water-labile CDI. Therefore, the suspending medium should be exchanged with other non-aqueous organic solvents. Although a previous study [29] reported a successful medium exchange by sonication of the freeze-dried nanowhiskers having surface sulfate groups in DMSO or DMF, the “charge-free” nanowhiskers in our study seemed to irreversibly aggregate during the sonication method, due to a lack of electrostatic repulsion. The authors examined a medium exchange of the suspension from water to DMAc through never-dried procedures described in the Experimental section, successfully yielding a nanowhisker suspension dispersed in DMAc. Although the obtained suspension indicated loose visible aggregates and did not show flow birefringence, they were non-sedimenting and could be used for further modification using mPEG1000-COOH and CDI.

Another component of the reaction, *i.e.*, mPEG1000 with terminal carboxyl groups, seemed to be available by the previously reported oxidation using TEMPO [47,48,49], a water-soluble radical oxidizing agent. However, the final step in the collection of the oxidized mPEG, *i.e.*, precipitation of the oxidized mPEG in cooled ethanol [47,48,49], may have been unsuccessful due to the relatively low molecular weight (1000) of PEG in the present study. This drawback was resolved by the use of silica gel catalysts carrying TEMPO moieties on their surface, instead of an aqueous TEMPO solution. The silica gel supported TEMPO catalysts was prepared according to a previous procedure [50] and can be successfully removed from the reaction mixture by a facile filtration after oxidation, as described in the Experimental section. Subsequent extraction with dichloromethane and rotary evaporation achieved successful recovery of the oxidized mPEG. The recovered silica gel supported TEMPO could be used in the next oxidation cycle. The carboxyl content of mPEG1000-COOH prepared by the first, second and third cycles of silica gel TEMPO were 1.12, 0.881 and 0.824 mmol/g, respectively, corresponding to 112%, 88.1% and 82.4% terminal oxidation. Terminal oxidation beyond 100% may be due to the presence of some dihydroxy PEG in the commercial mPEG reagent [51]. Although the reasons for lower oxidation ratio with repeated use of the silica gel TEMPO, in contrast to the previous results [50], are still unknown, it should be considered that some modifications of conditions, *i.e.*, increase in NaClO amount or reaction time, might be necessary in the repeated oxidation cycles. Because we did not perform further purification of mPEG1000-COOH, namely removal of unoxidized mPEG, the oxidation ratio in the present study should be estimated as at least 82%. Although we do not consider that the unoxidized mPEG, *i.e.*, mPEG1000-OH, is not involved in the subsequent grafting reactions, it may be bound to the surface of the nanowhiskers as well as mPEG1000-COOH, because one previous study [52] indicated a CDI-mediated binding (cross-linking) of two hydroxyls.

### 2.2. Qualitative Evaluation of Dispersion Stability of the PEG-Grafted Suspensions

**Figure 1:**
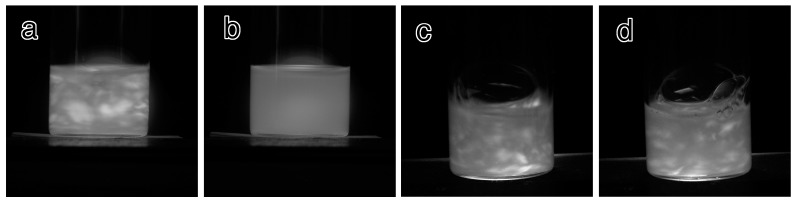
Appearances of the nanowhisker suspensions shaken between crossed polarizers. (**a**) Ungrafted sample suspended in water; (**b**) ungrafted sample in 0.1 M NaCl, (**c**) 1k-DMAc-100C-7d in water; and (**d**) 1k-DMAc-100C-7d in 0.1 M NaCl.

**Figure 2:**
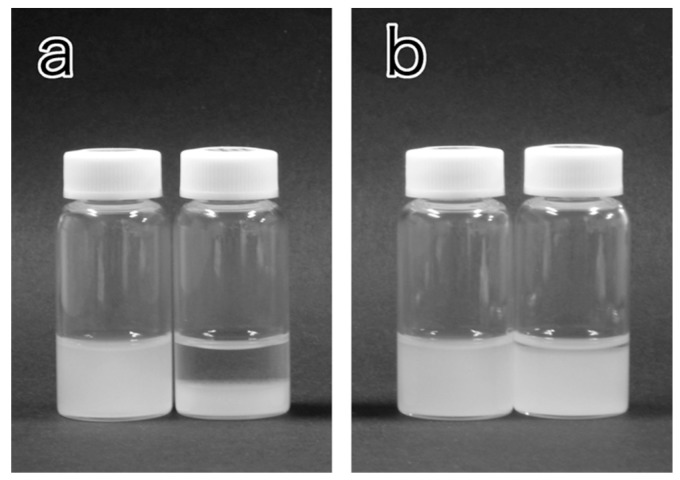
Appearances of the (**a**) ungrafted material and (**b**) 1k-DMAc-100c-7d after standing for 1 h. In both photographs, the NaCl concentrations in the left and right vials were 0 and 0.1 M, respectively. Nanowhisker content of the all samples was 0.1 wt %.

Various mPEG-grafted samples were prepared under the various conditions summarized in the Experimental Section. Prior to a detailed characterization, we first examined observation of their qualitative stability in the presence and absence of electrolyte. The indicators of suspension stability were two features, *i.e.*, flow birefringence of the sample and formation of precipitates after standing. While suspensions of well-dispersed nanowhiskers usually show remarkable flow birefringence because of their anisotropic morphology, formation of apparently isotropic aggregates eliminates their birefringent nature, and results in precipitation after standing. Table 1 summarizes the dispersion stability results of the suspensions in water or 0.1 M NaCl. The ungrafted sample was already known to be stable in water but lost its stability with the addition of a trace amount of electrolyte (a drop of 1 M KCl [44]) and sedimented. The current work also indicated the loss of flow birefringence and precipitation within 5 min of the ungrafted in 0.1 M NaCl. In contrast, none of the mPEG-grafted samples lost their flow birefringence and they were stable without precipitation at least for 1 h in 0.1 M NaCl. These results clearly show the improvements in dispersion stability by mPEG grafting. However, some of the mPEG-grafted samples, *i.e.*, 1k-DMAc-80C-1d, 1k-DMSO-80C-1d, 1k-DMAc-80C-3d and 1k-DMSO-80C-3d, sedimented after 24 h, even in water, in contrast to the stability of the ungrafted material in water. However, 1k-DMAc-100C-3d and 1k-DMAc-100C-7d in water and 1k-DMAc-100C-7d in 0.1 M NaCl still showed dispersion after 24 h. These results indicates that, while the former four samples formed aggregates over a long time scale, taking up to 24 h to sediment, the latter two acquired sufficient stability in the presence of 0.1 M NaCl through adequate levels of mPEG grafting. Figure 1 indicates flow birefringence of the ungrafted material and 1k-DMAc-100C-7d in water and 0.1 M NaCl. Appearances of the ungrafted material and 1k-DMAc-100C-7d with or without NaCl after standing for 24 h are shown in Figure 2. All the grafted samples tended to show considerable degree of aggregation/sedimentation in the presence of higher electrolyte concentration (for example, 0.5 M NaCl). The results indicate the less stability of the present grafted samples than that in our previous study [33], implying the improved stability of the latter aided by charge repulsions generated by residual surface carboxyl groups.

**Table 1:** Qualitative observation of flow birefringence and sedimentation for ungrafted material and mPEG-grafted nanowshiker suspensions prepared under various conditions.

Sample Name	Flow Birefringence ^a^		Dispersion Stability after 24 h ^b^	
	in Water	in 0.1 M NaCl	in Water	in 0.1 M NaCl
ungrafted	+	-	a	a
1k-DMAc-80C-1d	+	+	a	a
1k-DMSO-80C-1d	+	+	a	a
1k-DMAc-80C-3d	+	+	a	a
1k-DMSO-80C-3d	+	+	a	a
1k-DMAc-100C-3d	+	+	d	a
1k-DMAc-100C-7d	+	+	d	d ^c^

^a^ +: flow birefringent, -: no flow birefringence; ^b^ a: aggregated, d: dispersed; ^c^ Very thin supernatant layer was observed in some repeated trials (see Figure 2b for example).

Two samples showing higher levels of stability, *i.e.*, 1k-DMAc-100C-3d and 1k-DMAc-100C-7d, were subjected to the following further characterizations, including confirmation of ester formation by ATR-FTIR, evaluation of the amount of grafted mPEG and TEM observation.

### 2.3. Evaluation of Amount of Bound mPEG

As described in the Experimental Section, the amount of grafted mPEG1000 was determined by two methods; the one is measurement of weight increase after grafting, and the other is determination of weight decrease of the mPEG-grafted samples after alkali cleavage of the ester binding moiety and subsequent removal of mPEG by thorough washing. The employed conditions seem to be sufficient for cleave ester linkages compared with those in the typical ester hydrolyses, *i.e.*, at room temperature or higher, from 30 min to 24 h, at alkali concentrations of 0.1–2 M [53].

Table 2 summarizes the amount of mPEG1000 grafted onto the two samples, namely 1k-DMAc-100C-3d and 1k-DMAc-100C-7d, determined by the two methods described above. Values for both samples were 0.30–0.32 g/g cellulose, which were almost the same as that reported for an adduct of aminated mPEG1000 and carboxylated nanowhiskers in a previous study [33]. The results in Table 2 also confirm the comparability of the values determined by the different two methods. The ungrafted nanowhiskers were also treated with 0.5 M NaOH and subsequently washed to elucidate the effect of the alkali treatment scheme on the change in nanowhisker weight, giving a weight decrease of 0.03 g for 1 g of nanowhisker. Although this value may be negligible, the values determined by the alkali hydrolysis method can involve this level of deviation.

**Table 2:** The amount of grafted mPEG-COOH1000 in 1k-DMAc-100C-3d and 1k-DMAc-100C-7d, calculated from weight increase and weight loss after alkali hydrolysis.

Samples	The Amount of Grafted mPEG-COOH1000, g/g Cellulose	
	from Weight Increase	from Weight Loss after Alkali Cleavage
ungrafted	–	0.03
1k-DMAc-100C-3d	0.30	0.26
1k-DMAc-100C-7d	0.32	0.25

### 2.4. Confirmation of mPEG Binding by ATR-FTIR Spectrometry

**Figure 3:**
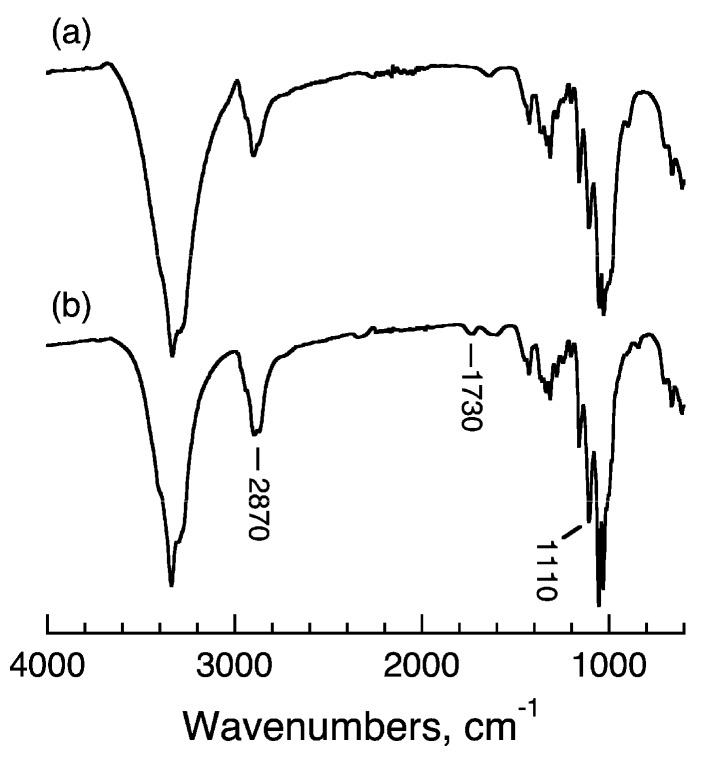
ATR-FTIR spectra of (**a**) ungrafted material and (**b**) 1k-DMAc-100c-7d.

ATR-FTIR spectra of the ungrafted material and 1k-DMAc-100C-7d are shown in Figure 3. Absorptions that appear in the spectrum of the ungrafted material are attributable to those of typical crystalline native cellulose [54]. On the other hand, increases in some absorption bands attributable to those of amorphous PEG, for example at 2870 and 1110 cm^−1^ [55], can be observed in the spectrum of 1k-DMAc-100C-7d, although they are obscured by overlap with the absorptions of cellulose. The latter spectrum also shows a remarkable absorption at 1730 cm^−1^, resulting from the *υ*_C=O_ of ester linkages between mPEG1000 and the surface hydroxyl of cellulose. This clearly confirms the covalent binding of mPEG1000 onto the nanowhisker surface via ester formation, rather than mere surface absorption. The absorptions characteristic of crystalline PEG [55] are not observed in the spectrum of 1k-DMAc-100C-7d, suggesting the amorphous or independent nature of the grafted mPEG1000. Because a previously reported sample with similar levels of mPEG1000 grafting [33] also showed non-crystallinity in the grafted mPEG, the grafting density of mPEG1000 in the present study and ref. 33 was insufficient for crystallization on the surface. An almost identical tendency was observed for 1k-DMAc-100C-3d (data not shown).

### 2.5. Transmission Electron Microscopy (TEM)

**Figure 4:**
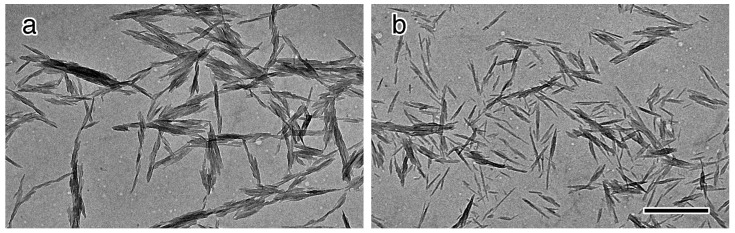
Electron micrographs of (**a**) ungrafted material and (**b**) 1k-DMAc-100c-7d. The scale bar is 500 nm.

Figure 4 shows electron micrographs of the ungrafted and 1k-DMAc-100C-7d. Both micrographs show similar size and shape of the nanowhiskers, similar to those in previous studies [16,33], *i.e.*, 5–10 nm width and 100–150 nm length, suggesting no significant effect of the mPEG grafting reaction on the morphology of the nanowhiskers. While the ungrafted material showed significant aggregation due to a lack of surface repulsion, the micrograph of 1k-DMAc-100C-7d showed somewhat improved dispersion, whereby even small bundles of several nanowhiskers still remained.

## 3. Experimental Section 

### 3.1. Materials

Purified fibrous cotton (CF11, Whatman International Ltd., Maidstone, Kent, UK) was used as starting cellulose. 1,1'-carbonyldiimidazole (CDI) and the free radical form of 4-oxo TEMPO (4-oxo-2,2,6,6-tetramethylpiperidine-1-oxyl) was purchased from Tokyo Chemical Industry Co. Ltd. (Tokyo, Japan) and Wako Chemical Ltd. (Miyazaki, Japan), respectively. 3-Aminopropyl-functionalized silica gel (40–63 µm, ~1 mmol/g NH_2_ loading) and polyethylene glycol 1000 monomethyl ether (mPEG1000) were purchased from Sigma-Aldrich Co. (St. Louis, MO, USA). Other reagents were from Wako Pure Chemical Industries, Ltd. (Osaka, Japan). Dimethylacetamide (DMAc) and dimethyl sulfoxide (DMSO) were dehydrated by reduced distillation after treatment with CaH_2_. Dry methanol was obtained after distillation following treatment with magnesium and iodine. Other reagents were used without further purification.

### 3.2. Preparation of Cellulose Nanowhiskers

An aqueous suspension of “charge-free” cellulose nanowhiskers was prepared according to previous reports [33,43,44]. Briefly, air-dried CF11 (10 g) was treated with 2.5 M HCl (100 mL) at 105 °C for 15 min, followed by successive washing by filtration using deionized water until neutrality. The hydrolyzed sample was suspended in deionized water to form a 5%–10% slurry and homogenized with a Waring-type blender for 30 min. The non-sedimenting nanowhisker suspension was collected as a turbid supernatant through repeated centrifugation cycles (1600 *g*, 5 min) of the homogenized sample and further dialyzed against deionized water to give a 0.5%–1% nanowhisker suspension. The initial ungrafted nanowhiskers and their suspension are designated as “ungrafted” hereafter. Yield from starting CF11 was around 40%–50%.

### 3.3. Synthesis of Terminal-Carboxylated mPEG (mPEG-COOH) via Oxidation Using Silica Gel Supported TEMPO

Terminal hydroxyl groups of mPEG1000 were oxidized into carboxyl groups according to previously published procedures [47,48,49], with the modification of using silica gel particles carrying the TEMPO moieties instead of aqueous TEMPO solution. For this purpose, silica gel supported TEMPO was prepared via reductive amination of the amino groups on the silica gel and 4-oxo TEMPO, according to the literature [50]. The silica gel product carrying TEMPO (1.00 g) was dispersed in a solution of mPEG1000 (10.0 g, 10.0 mmol -OH) and sodium bromide (100 mg, 0.972 mmol) in deionized water (100 mL). After adjustment of the pH to around 6 with 1 M HCl, sodium hypochlorite (NaClO) solution (21.1 mL, adjusted to contain 40.0 mmol of NaClO) was slowly added. The reaction mixture was stirred for 1 h at room temperature, while keeping the pH around 7–8 with dropwise addition of 2 M NaOH aqueous solution. After oxidation, 10 mL of ethanol was added to quench the oxidation through consumption of excess NaClO. The silica-gel catalyst was removed by filtration. The obtained filtrate was adjusted to pH 1 by addition of 1 M HCl, followed by extraction of oxidized mPEG with dichloromethane (100 mL × 3) and concentration by rotary evaporation. Yield of the oxidized mPEG1000, named mPEG1000-COOH hereafter, was 92.7%. The conversion ratio of the terminal hydroxyl groups was determined by pH titration using 0.1 M aqueous NaOH and a phenolphthalein indicator.

### 3.4. Grafting of mPEG-COOH onto Surfaces of Cellulose Nanowhiskers via CDI-Mediated Esterification

Prior to mPEG grafting, the cellulose nanowhiskers were dispersed in DMAc by a solvent exchange process to obtain a dehydrated suspension, as follows; the aqueous suspension of the ungrafted nanowhiskers (83.3 g, 1.20 wt % solid content, containing 1.00 g nanowhiskers) were mixed with three times the volume of acetone, followed by precipitation of nanowhiskers by three cycles, including centrifugation (1600 *g*, 10 min), discarding of supernatant and further mixing with fresh acetone. The obtained pellet of acetone-containing nanowhiskers were mixed with dehydrated DMAc (40 mL) and treated with a rotary evaporator (up to 60 °C, *ca.* 30 min) to evaporate residual acetone and yield a suspension dispersed in DMAc. The precise solid content of the obtained DMAc nanowhisker suspension was determined by mixing a weighed amount of the suspensions (typically around 1 g) with methanol (*ca.* 50 mL), washing the nanowhiskers with methanol by repeated centrifugation and weighing after drying the pellet.

Another aliquot of DMAc solution (5 mL) containing mPEG1000-COOH (1.00 g, 1.00 mmol -COOH) and CDI (0.200 g, 1.23 mmol) was stirred for 2 h at room temperature, and subsequently mixed with 20 mL of the DMAc nanowhisker suspension (containing 0.500 g of nanowhiskers, assuming no handling loss). The mixture was stirred for various periods of time at various temperatures, summarized in Table 3, followed by dilution with deionized water and thorough dialysis against deionized water (using regenerated cellulose membrane with MWCO = 12,000–14,000, typically over 3 days with exchanges of water twice a day), to yield PEG-grafted samples. The samples were designated using the molecular weight of PEG, reaction medium, reaction temperature and reaction time. For example, 1k-DMAc-80C-1d was prepared by a reaction using nanowhiskers and mPEG1000-COOH (= 1k) at 80 °C for 1 day.

**Table 3:** Reaction conditions used to prepare mPEG-grafted nanowhisker suspensions.

Sample Name	Reaction Medium	Reaction Temperature, °C	Reaction Time, day(s)	Weight of Nanowhiskers, g	Weight of mPEG1000–COOH, g (mmol)	Weight of CDI, g (mmol)
1k-DMAc-80C-1d	DMAc	80	1	0.464	1.0 (1.00)	0.200 (1.23)
1k-DMSO-80C-1d	DMSO	80	1	0.450	1.0 (1.00)	0.200 (1.23)
1k-DMAc-80C-3d	DMAc	80	3	0.394	0.5 (0.500)	0.100 (0.616)
1k-DMSO-80C-3d	DMSO	80	3	0.424	0.5 (0.500)	0.100 (0.616)
1k-DMAc-100C-3d	DMAc	100	3	0.500	0.5 (0.500)	0.100 (0.616)
1k-DMAc-100C-7d	DMAc	100	7	0.500	0.5 (0.500)	0.100 (0.616)

The weight ratio of nanowhiskers to mPEG1000-COOH was adjusted to 0.5 or 1, although some deviations were observed because of difficulties in keeping the concentrations of the DMAc nanowhisker suspension at the final evaporation state. The precise amounts of nanowhiskers subjected to the reactions are described in Table 3, although their deviation appears to be negligible. In all cases, a 1.2 equivalent molar amount of CDI against that of mPEG (calculated only from weight, without considering terminal oxidation ratio) was added, as also shown in Table 3.

### 3.5. Qualitative Evaluation of Dispersion Stability of the PEG-Grafted Suspensions

The ungrafted and the PEG-grafted suspensions were adequately diluted with deionized water and/or 1 M NaCl aqueous solution to give equal nanowhisker content of 0.1% (excluding the weight of PEG in the cases of the PEG-grafted samples, by considering the weight increase, as in the next section) and NaCl concentrations of 0 or 0.1 M. The suspensions were observed between crossed polarizers to check the appearances of flow birefringence. Further, all of the suspensions were allowed to stand at room temperature for up to 24 h for visual observation of precipitation. 

### 3.6. Evaluation of Amount of Grafted PEG

Evaluation of the amount of grafted PEG was performed by two methods. The first method was by the increase in sample weight by determining the concentration of suspensions before and after PEG grafting. From preliminary experiments, the final dialysis step in the preparation was found to completely remove unreacted mPEG1000-COOH. The other method was based on alkali hydrolysis of the ester moiety between nanowhiskers and mPEG1000. The PEG-grafted samples with a known value of solid content (*i.e.*, known weight of nanowhiskers + PEG) were mixed with an equal amount of 1 M aqueous NaOH, followed by overnight stirring at room temperature. Through this alkali treatment, the ester moiety between nanowhiskers and mPEG1000 was believed to be completely hydrolyzed, whereas the nanowhiskers remained intact. The hydrolyzed samples were carefully neutralized with 3 M HCl, washed with deionized water by repeated centrifugation, and dried to yield the weight of “bare” nanowhiskers. The decrease in weight was determined by subtraction from the initial weight. The values of the grafted PEG weight determined by these two methods were converted into the PEG weight grafted to 1 g of nanowhiskers.

### 3.7. Measurements

Size and shape of the nanowhiskers were observed by transmission electron microscopy (TEM). A drop of very dilute suspension was deposited on a TEM grid coated with a Formvar film, which was pretreated with 0.2% aqueous bacitracin solution. The dried grid was observed using a JEOL JEM-2100 instrument (JEOL Ltd., Tokyo, Japan) at 80 kV using defocus contrast technique. Attenuated total reflection Fourier-transform infrared (ATR-FTIR) spectra of the freeze-dried samples were recorded on a Shimadzu IRPrestage-21 spectrometer with a DurasampleIR II ATR accessory (SensIR Technologies, Danbury, CT, USA) at a resolution of 4 cm^−1^ using 32 scans.

## 4. Conclusions 

Successful grafting of carboxylated mPEG1000 onto “charge-free” nanowhiskers was achieved in the present study via CDI-mediated esterification. Grafting of mPEG1000 was clearly indicated by outstanding improvements in dispersion stability of the mPEG-grafted nanowhiskers in the presence of NaCl and confirmation of ester formation by ATR-FTIR. The amount of grafted mPEG was estimated as *ca.* 0.3 g/g cellulose by both weight increase and alkali cleavage. TEM observations confirmed that the morphology of the nanowhiskers remained unchanged.

More detailed investigations into effect of mPEG length on changes in PEG grafting amount and dispersion stability should be performed in a future study. Higher mPEG grafting using more active electrophilic groups such as carboxylic acid halides will be examined. Dispersion of the mPEG-grafted nanowhiskers in organic solvents should be also examined for future creation of novel nanocomposites with organosoluble polymer matrices.
